# Supplementary material for: Effect of an enhanced public health contact tracing intervention on the secondary transmission of SARS-CoV-2 in educational settings: The four-way decomposition analysis
Source: eLife. 2024 Feb 28;13:e85802. doi: 10.7554/eLife.85802 (PMC10901504; doi:10.7554/eLife.85802)
Supplement: Supplementary file 1. [file elife-85802-supp1.docx]

Supplementary file 1. Description of effects in the decomposition mediation analysis

| **Effect** | **Definition** | **Interpretation** |
| --- | --- | --- |
| Total effect | Overall effect of the intervention on the outcome | The overall effect of the prompt contact tracing on the secondary transmission. |
| Controlled direct effect (CDE) | Intervention effect that is neither due to mediation nor interaction, i.e., when the mediator is fixed at an arbitrary value | Effect of the prompt contact tracing on the secondary transmission when the source of infection of the index case is known or unknown. |
| If M=0 | Intervention effect in the absence of mediator | Effect of the intervention on the secondary transmission if the source of infection of the index case was unknown. |
| If M=1 | Intervention effect in the presence of mediator | Effect of the intervention on the secondary transmission if the source of infection of the index case was known. |
| Pure natural indirect effect (NIE) | Intervention effect only due to mediation, i.e., the effect of the mediator in the absence of intervention | Effect of the known source of infection of the index case on the secondary transmission in the period before intervention. |
| Mediated interaction  (INT_MED_) | Intervention effect only due to both mediation and intervention, i.e., allowing the levels of the mediator to vary across the compared intervention groups | Interactive effect between the prompt contact tracing operating through the known source of infection of the index case, allowing the levels of the source of infection of the index case to vary before and after intervention. |
| Reference interaction  (INT_REF_) | Intervention effect only due to interaction and not mediation, i.e., setting the mediator to be either present or absent | Effect of the interaction between prompt contact tracing and source of infection of the index case on the secondary transmission if the source of infection of the index case was known or unknown. |
| If M=0 | Intervention effect only due to interaction in the absence of mediator | Effect of the interaction between intervention and source of infection of the index case on the secondary transmission if the source of infection of the index case was unknown. |
| If M=1 | Intervention effect only due to interaction in the presence of mediator | Effect of the interaction between intervention and source of infection of the index case on the secondary transmission if the source of infection of the index case was known. |
