# Supplementary material for: Effect of an enhanced public health contact tracing intervention on the secondary transmission of SARS-CoV-2 in educational settings: The four-way decomposition analysis
Source: eLife. 2024 Feb 28;13:e85802. doi: 10.7554/eLife.85802 (PMC10901504; doi:10.7554/eLife.85802)
Supplement: Supplementary file 2. [file elife-85802-supp2.docx]

**Supplementary file 2.** Sample Stata code for mediation analysis using med4way

NOTE: the highlighted (in bold colour) are variables names, lists, or parameters to be chosen by the user.

* When mediator is equal to 1 (known contact):

Outcome: number of secondary cases (continuous)

Exposure: intervention (binary)

Mediator: source of infection (binary)

Covariates: type of school (kindergarten), class size (<21 students), type of index case (student)

med4way **outcome exposure** **mediator covariates**, a0(0) a1(1) m(0) yreg(negbin) mreg(logistic)  fulloutput c(1 0 0)

* When mediator is equal to 0 (unknown contact)

Outcome: number of secondary cases (continuous)

Exposure: intervention (binary)

Mediator: source of infection (binary)

Covariates: type of school (kindergarten), class size (<21 students), type of index case (student)

med4way **outcome exposure** **mediator covariates**, a0(0) a1(0) m(0) yreg(negbin) mreg(logistic)  fulloutput c(1 0 0)
